# Characterization of Pectoralis Major Muscle Satellite Cell Population Heterogeneity, Macrophage Density, and Collagen Infiltration in Broiler Chickens Affected by Wooden Breast

**DOI:** 10.3389/fphys.2020.00529

**Published:** 2020-05-27

**Authors:** Tamara Z. Ferreira, Liris Kindlein, Joshua J. Flees, Lauren K. Shortnacy, Sergio L. Vieira, Vladimir P. Nascimento, Kathryn J. Meloche, Jessica D. Starkey

**Affiliations:** ^1^Department of Preventative Veterinary Medicine, Federal University of Rio Grande do Sul, Porto Alegre, Brazil; ^2^Department of Poultry Science, Auburn University, Auburn, AL, United States; ^3^Department of Animal Science, Federal University of Rio Grande do Sul, Porto Alegre, Brazil; ^4^Department of Animal Medicine, Federal University of Rio Grande do Sul, Porto Alegre, Brazil

**Keywords:** Wooden Breast, muscle satellite cell, myogenic stem cell, macrophage, collagen infiltration, broiler chicken

## Abstract

Muscle satellite cells (MSCs) are myogenic stem cells that play a critical role in post-hatch skeletal muscle growth and regeneration. Activation of regeneration pathways to repair muscle fiber damage requires both the proliferation and differentiation of different MSC populations as well as the function of resident phagocytic cells such as anti-inflammatory and pro-inflammatory macrophages. The Wooden Breast (WB) phenotype in broiler chickens is characterized by myofiber degeneration and extensive fibrosis. Previous work indicates that the resident MSC populations expressing the myogenic regulatory factors, Myf-5 and Pax7 are larger and more proliferative in broilers severely affected with WB vs. unaffected broilers. To further characterize the cellular and molecular changes occurring in WB-affected muscles, samples from pectoralis major (PM) muscles with varying severity of WB (WB score 0 = normal; 1 = mildly affected; 2 = severely affected) were collected at 25 and 43 days post-hatch (*n* = 8 per score per age) and processed for cryohistological and protein expression analyses. Collagen per field and densities of macrophages and MyoD+, Myf-5+, and Pax7+ MSC populations were quantified on immunofluorescence-stained cryosections. Relative collagen protein expression was quantified by fluorescent Western Blotting. In both 25 and 43-days-old broilers, the proportion of collagen per field (*P* ≤ 0.021) and macrophage density (*P* ≤ 0.074) were greater in PM exhibiting severe WB compared with normal. At day 43, populations of MyoD+, Myf-5+:MyoD+ MSC were larger and relative collagen protein expression was greater in WB-affected vs. unaffected broilers (*P* ≤ 0.05). Pax7+ MSC relative to total cells was also increased as WB severity increased in 43-days-old broilers (*P* ≤ 0.05). Densities of Myf-5+ (*P* = 0.092), MyoD+ (*P* = 0.030), Myf5+:MyoD+ (*P* = 0.046), and Myf-5+:MyoD+:Pax7+ (*P* = 0.048) MSC were greater in WB score 1 birds compared with WB score 0 and 2 birds. Overall, alterations in the resident MSC and macrophage populations and collagen protein content were observed in WB-affected muscle. Further investigation will be required to determine how these changes in cell population kinetics and local autocrine and paracrine signaling are involved in the apparent dysregulation of muscle maintenance in WB-affected broilers.

## Introduction

Both global and domestic demand for chicken meat continue to steadily increase making it arguably the most important meat protein source in the world. In the United States (US), the demand is greatest for high quality, white (breast, *pectoralis major* muscle, PM) meat. To meet this demand, the commercial poultry industry has placed tremendous genetic selection pressure on breast meat yield, growth rate, and feed efficiency traits and has made remarkable improvements over the last 40 years (Zuidhof et al., 2014). Unfortunately, along with those tremendous improvements has come a severe meat quality defect, the cause of which has yet to be elucidated. The defect referred to as both Woody Breast and Wooden Breast (WB) is characterized by visible bulging and extreme palpable hardness of the PM. The WB phenotype has been characterized by histopathologists as a degenerative myopathy that manifests in fast-growing, high-meat-yielding broiler chickens and results in myofiber necrosis, excessive fibrosis, and immune cell infiltration inside the perimysium (Petracci and Cavani, 2012; Sihvo et al., 2014; Velleman and Clark, 2015). The safety and wholesomeness of the product are not negatively impacted, but the poultry industry nevertheless continues to incur large economic losses due to decreased product acceptability and functionality (Kuttappan et al., 2016; Soglia et al., 2016; Tasoniero et al., 2016; Tijare et al., 2016). From a product quality standpoint, the WB phenotype has been reasonably well-characterized. However, to date, the specific cellular and molecular mechanisms that lead to the development of WB are still unclear.

Skeletal muscle satellite cells (MSCs) play a critical role in post-hatch broiler chicken skeletal muscle fiber hypertrophic growth and are essential for normal muscle maintenance and repair (Armand et al., 1983; Yablonka-Reuveni et al., 1987). The rapid increase in the muscle fiber cross-sectional area (CSA) that occurs in broiler chickens during the normal 4 to 10-week rearing period is mediated by extensive MSC proliferation, differentiation (accompanied by withdrawal from the cell cycle), and fusion with the existing muscle fibers (Campion, 1984; Hutton et al., 2014). Thus far, the relationship between MSC function in rapidly-growing, high-yielding broilers and the development of the WB myopathy has been largely unexplored. However, it is known that the activation of muscle repair and regeneration pathways requires both the proliferation and differentiation of different MSC populations as well as the function of resident phagocytic cells such as anti-inflammatory and pro-inflammatory macrophages, which produce cytokines known to impact MSC function (Cantini et al., 1994). The relationships among the different MSC populations and macrophages and how they relate to collagen infiltration in WB-affected muscle are unclear. Therefore, the objective of this work was to explore the changes in the heterogeneity of myogenic regulatory factor (MRF) expression in MSC populations and to quantify macrophage densities and collagen protein expression in broilers with increasing severity of WB over time.

## Materials and Methods

### Bird Husbandry

The Auburn University Institutional Animal Care and Use Committee approved the use of live birds and all procedures performed in this experimental protocol (PRN 2016-2829). As previous described by Meloche et al. (2018a), day-old, male, Yield Plus × Ross 708 broiler chicks were obtained from a commercial hatchery (*n* = 480, Aviagen Group, Huntsville, AL, United States). Chicks were vaccinated for Newcastle disease, Marek’s disease, and infectious bronchitis at the hatchery. From 1 to 6 days of age, chicks were housed in groups of 8 in raised floor pens (0.03 m^2^/bird) bedded with new pine shavings, containing individual feeders, 2 nipple waterers per pen located in a solid-sided, temperature-controlled, dehumidified research facility. At 7 days of age, all chicks were weighed and the lower and upper 12% of the BW range were excluded. The remaining 360 chicks were identified with wing bands and allocated by weight into the individual-housing pens (0.20 m^2^/bird). Ambient temperature was set to 33°C on day 0 and reduced to maintain comfort until day 43. Birds were exposed to a photoperiod of 23 h from placement to 7 days of age, followed by a photoperiod of 18 h for the remainder of the experiment. Light intensity was set at 30 lux from 1 to 7 days of age, 10 lux from 8 to 14 days of age, 5 lux from 15 to 24 days of age, and 3 lux from 25 to 43 days of age. Light intensity settings were verified at bird level (30 cm) using a photometric sensor with National Institute of Standards and Technology-traceable calibration (Model No. 403125, Extech Instruments, Waltham, MA, United States) for each intensity adjustment. All birds consumed fresh water and feed offered in four dietary phases on an *ad libitum* basis. Birds whose samples were chosen for this experiment all consumed the same corn and soybean meal-based Control grower 2 diet (formulated at 100% of primary breeder nutrient recommendations for digestible Lys) from days 15 to 25 which is described in detail in Table 1 of Meloche et al. (2018a).

**TABLE 1:** Effect of Wooden Breast on density, relative density, and heterogeneity of *Pectoralis major* muscle satellite cell populations in broiler chickens at 25 days of age.

**Cell population^2,3^**	**Wooden Breast Score^1^**			**SEM^4^**	***P*-value**
	**Normal (0)**	**Mild (1)**	**Severe (2)**		
**Density, cells per mm^2^**					
Myf-5−:MyoD−:Pax7− (non-myogenic)	412.69	521.44	547.55	107.92	0.650
Myf-5+	106.63	84.75	139.37	19.46	0.161
MyoD+	16.38	16.50	12.00	6.44	0.855
Pax7+	60.25	60.63	78.75	13.85	0.567
Myf-5+:MyoD+	37.38	39.50	35.00	13.31	0.972
Myf-5+:Pax7+	11.25	17.25	7.50	6.50	0.573
MyoD+:Pax7+	6.75	12.75	15.75	5.06	0.454
Myf-5+:MyoD+:Pax7+	18.00	21.00	45.50	9.59	0.109
**Relative density, % of total DAPI+**					
Myf-5−:MyoD−:Pax7− (non-myogenic)	61.69	67.46	62.14	0.05	0.644
Myf-5+	15.94	10.96	15.82	0.03	0.459
MyoD+	2.45	2.14	1.36	0.01	0.487
Pax7+	9.01	7.84	8.94	0.01	0.825
Myf-5+:MyoD	5.58	5.11	3.97	0.02	0.825
Myf-5+:Pax7+	1.68	2.23	0.85	0.01	0.569
MyoD+:Pax7+	1.01	1.65	1.79	0.01	0.649
MyoD+:Myf-5+: Pax7+	2.69	2.72	5.16	0.01	0.173
**Relative density, % of total myogenic**					
Myf-5+	41.59	33.65	41.78	0.05	0.374
MyoD+	6.38	6.55	3.59	0.02	0.537
Pax7+	23.50	24.07	23.60	0.06	0.997
Myf-5+:MyoD+	14.58	15.68	10.49	0.04	0.587
Myf-5+:Pax7+	4.39	6.85	2.25	0.02	0.416
MyoD+:Pax7+	2.63	5.06	4.72	0.02	0.593
Myf-5+:MyoD+: Pax7+	7.02	8.34	13.64	0.03	0.104

*^1^The *Pectoralis major* muscles of all birds were visually assessed and scored on a 3-point scale (0 = normal; 1 = mild; 2 = severe) for Wooden Breast (*n* = 8 per score). ^2^DAPI = 4′6-diamindino-phenylindole nuclear counterstain. All subsequent cell populations may be assumed to be DAPI+. ^3^Total describes every cell positive for the specific immunofluorescence target protein, regardless of the status of other targets; Non-myogenic describes cells not positive for Myf-5, Pax7, MyoD, or any combination thereof; Myogenic describes cells positive for Myf-5, Pax7, MyoD, or any combination thereof. ^4^SEM = highest standard error of the pair-wise mean comparisons. ^*a,b*^Means within the same row with different superscripts differ *P* ≤ 0.05. ^*x,y*^Means within the same row with different superscripts differ 0.0501 ≤ *P* ≤ 0.10.*

### Wooden Breast Scoring and Muscle Sample Collection

At days 25 and 43 post-hatch, birds (*n* = 50 per day) were euthanized by CO_2_ asphyxiation followed immediately by cervical dislocation and samples (≈1.25 cm × 0.635 cm × 0.635 cm) from the anteroventral portion of the left PM muscle were excised and processed for cryohistological immunofluorescence staining analyses according to procedures adapted from Hutton et al. (2014) and described in Meloche et al. (2018a). Muscle samples immediately adjacent those taken for cryohistology were also collected from each bird, snap frozen in liquid nitrogen, and stored at −80°C prior to analysis for protein expression by quantitative, fluorescent Western Blotting as described below. Samples for this experiment (*n* = 8 per WB score per day) were obtained from birds with WB scores of 0, 1, and 2 on a 3-point scale (WB score 0 = normal, 1 = mildly affected, and 2 = severely affected) as determined by visual evaluation and physical palpation of the PM muscles at sampling. All PM muscles were scored by the same evaluator and considered “normal” if there was no palpable hardness in any of the PM, “mild” if palpable hardness was present in less than half the total PM muscle surface area, and “severe” if it exceeded this limit.

### Cryohistological Immunofluorescence Analysis

Samples stored at −80°C prior to analysis were warmed to −20°C for at least 16 h and subsequently cryosectioned using a Leica CM 1950 cryomicrotome. Serial 5-μm-thick, cross-sections were cut from each PM sample, mounted on positively charged glass slides (VWR International, Westchester, PA, United States), and stored at 4°C before immunofluorescence staining as described in Meloche et al. (2018a). All slides were briefly counter-stained with 4′,6-diamidino-phenylindole (DAPI; 1 μg per mL; VWR International) to facilitate determination of total nuclear density. Control cryosections processed as described above, but without the addition of either primary or secondary antibodies, were used to ensure that no fluorescence signal beyond natural autofluorescence was observed for the selected combination of antibodies confirmed to be cross-reactive with chicken described below.

**FIGURE 1:**
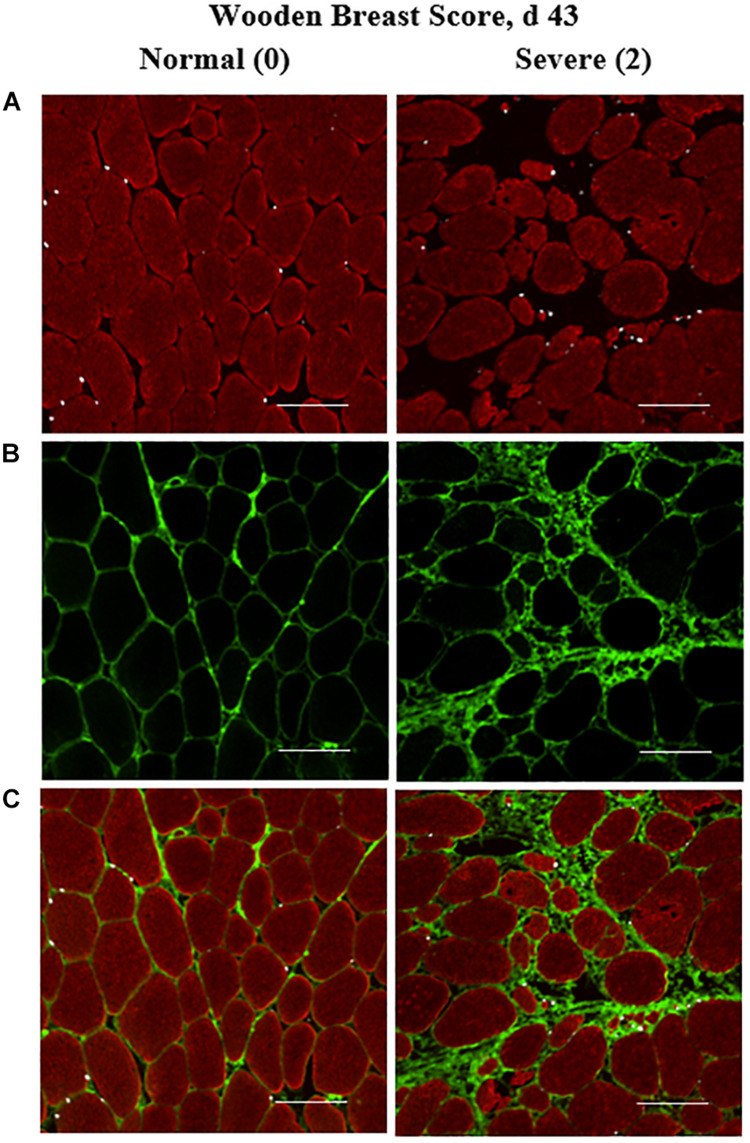
Effect of Wooden Breast (WB) on *pectoralis major* muscle satellite cell (MSC) populations and collagen infiltration 43 days post-hatch in broiler chickens. **(A–C)** Shows a representative cryohistology immunofluorescence staining images for sarcomeric myosin (red), collagen (green), and Pax7+ MSC (white) among normal (WB score 0) and severely affected (WB score 2) PM muscle at 43 days of age (*n* = 8 per score). **(A)** Demonstrates the obvious myofiber disorganization and degeneration (red) as well as the increase in the density of Pax7+ MSC (white) in the WB-affected muscle compared with normal muscle. **(B,C)** Illustrate the clear increases in collagen infiltration (green) in the WB-affected muscle compared with normal. Scale bar = 100 μm.

Immunofluorescence-stained cryohistological slides were imaged at 100-fold and 200-fold magnification with an inverted fluorescence microscope (Nikon Eclipse, Ti-U; Nikon Instruments, Inc., Melville, NY, United States) fitted with a UV light source (Nikon Intensilight). Images were captured and analyzed using an Evolve 512 EMCCF camera (Photometrics, Tucson, AZ, United States) and Elements imaging software (Nikon Instruments, Inc.). A representative digital image at both magnifications was captured from each slide (2 slides per bird). Slides were simultaneously immunofluorescence-stained for the nuclear MRF MSC markers, Myf-5, MyoD, and Pax7 for determination of MSC population densities and heterogeneity of MRF expression, The MSC populations (Myf-5+, MyoD+, Pax7+, Myf-5+: MyoD+, Myf-5+: Pax7+, MyoD+:Pax7+, and Myf-5+:MyoD+:Pax7+) were enumerated in the 200-fold magnification images and their densities expressed on a per mm^2^ basis (Tables 1, 2). All cell populations enumerated were also DAPI+ in addition to their immunofluorescence profile. The total number of DAPI+ nuclei per image was determined in each image as a measure of nuclear density and to determine Total DAPI+ cells. Any cells positive for Myf-5, MyoD, Pax7 or any combination thereof were considered myogenic and those not positive for any of the target antigens were considered non-myogenic using antibodies previously validated for cross-reactivity with chicken (Yablonka-Reuveni, 1995; Day et al., 2009; Tejeda et al., 2019). Additional serial slides from 43-days-old birds were immunofluorescence-stained for sarcomeric myosin, collagen, and Pax7 to facilitate the visual illustration of the extensive collagen infiltration and increases in the local MSC populations observed in PM muscles severely affected with WB compared with those receiving normal WB scores (Figures 1A–C). The proportion of collagen fluorescence in each image was determined using the binary component in Elements software as previously reported by Murphy et al. (2011). Subsequently, additional slides with serial cryosections to those stained for MRF heterogeneity were immunofluorescence-stained from each bird and the 200-fold digital images were used to determine the proportion of collagen per image and density (per mm^2^) of the total macrophages, including both pro and anti-inflammatory populations using a general leukocyte/macrophage marker previously validated for use in chickens Mast et al. (1998) (Figures 3A–C).

**TABLE 2:** Effect of Wooden Breast on density, relative density, and heterogeneity of *Pectoralis major* muscle satellite cell populations in broiler chickens at 43 days of age.

**Cell population^2,3^**	**Wooden Breast Score^1^**			**SEM^4^**	***P*-value**
	**Normal (0)**	**Mild (1)**	**Severe (2)**		
**Density, cells per mm^2^**					
Myf-5-:MyoD−:Pax7− (non-myogenic)	267.43	308.41	403.76	56.25	0.236
Myf-5+	123.62^*x*^	187.12^*y*^	121.38^*x*^	22.83	0.092
MyoD+	3.75^*a*^	30.63^*b*^	9.00^*a*^	7.06	0.032
Pax7+	13.50^*x*^	26.25^*xy*^	52.87^*y*^	11.43	0.067
Myf-5+:MyoD+	58.13^*a*^	177.13^*b*^	57.37^*a*^	36.38	0.046
Myf-5+:Pax7+	0.75	7.50	3.75	2.79	0.252
MyoD+:Pax7+	10.50	12.00	3.00	4.08	0.269
Myf-5+:MyoD+:Pax7+	30.00^*a*^	72.37^*b*^	41.87^*ab*^	11.63	0.048
**Relative density, % of total DAPI+**					
Myf-5−:MyoD−:Pax7− (non-myogenic)	52.71^*ab*^	37.57^*a*^	58.29^*b*^	0.06	0.050
Myf-5+	24.37	22.80	17.52	0.04	0.272
MyoD+	0.74^*a*^	3.73^*b*^	1.29^*a*^	0.01	0.033
Pax7+	2.67^*a*^	3.19^*a*^	7.63^*b*^	0.01	0.042
Myf-5+:MyoD+	11.46	21.58	8.28	0.05	0.115
Myf-5+:Pax7+	0.15	0.92	0.54	0.01	0.452
MyoD+:Pax7+	2.07	1.46	0.43	0.01	0.121
Myf-5+:MyoD+:Pax7+	5.91	8.82	6.05	0.01	0.243
**Relative density, % of total myogenic**					
Myf-5+	51.54	36.51	42.02	0.06	0.174
MyoD+	1.56	5.98	3.12	0.01	0.105
Pax7+	5.63^*ab*^	5.12^*a*^	18.30^*b*^	0.03	0.029
Myf-5+:MyoD+	24.23	34.56	19.86	0.06	0.191
Myf-5+:Pax7+	0.31	1.46	1.29	0.01	0.702
MyoD+:Pax7+	4.38^*a*^	2.34^*ab*^	1.04^*b*^	0.01	0.085
Myf-5+:MyoD+:Pax7+	12.51	14.12	14.50	0.03	0.864

*^1^The *Pectoralis major* muscles of all birds were visually assessed and scored on a 3-point scale (0 = normal; 1 = mild; 2 = severe) for Wooden Breast (*n* = 8 per score). ^2^DAPI = 4′6-diamindino-phenylindole nuclear counterstain. All subsequent cell populations may be assumed to be DAPI+. ^3^Total describes every cell positive for the specific immunofluorescence target protein, regardless of the status of other targets; Non-myogenic describes cells not positive for Myf-5, Pax7, MyoD, or any combination thereof; Myogenic describes cells positive for Myf-5, Pax7, MyoD, or any combination thereof. ^4^SEM = highest standard error of the pair-wise mean comparisons. ^*a,b*^Means within the same row with different superscripts differ *P* ≤ 0.05. ^*x,z*^Means within the same row with different superscripts differ 0.0501 ≤ *P* ≤ 0.10.*

Primary antibodies utilized were as follows: rabbit IgG Type-1, α1 Collagen [Cat. No. sc-8784-R, 1:1,500 dilution; Santa Cruz Biotechnology, Santa Cruz, CA (SCB)]; mouse IgG1 Monocyte/Macrophage (Cat. No. sc-52603, 1:750 dilution; SCB); mouse IgG2b MyoD (Cat. No. sc-377460, 1:2,000 dilution; SCB); rabbit IgG Myf-5 (Cat. No. sc-302, 1:100 dilution; SCB), mouse IgG2b sarcomeric myosin (Cat. No. MF20, 1:10 dilution, Developmental Studies Hybridoma Bank (DSHB), Iowa City, IA, United States) and mouse IgG2b Pax7 (Cat. No. PAX7, 1:10 dilution, DSHB). Secondary antibodies (1:1,000 dilution) used for detection of the primary antibodies were as follows: AlexaFluor 488 Goat anti-rabbit IgG (H+L), AlexaFluor 546 Goat anti-mouse IgG1, AlexaFluor 633 Goat anti-mouse IgG2b, AlexaFluor 546 Goat anti-mouse IgG2b, AlexaFluor 633 Goat anti-mouse IgG1, and AlexaFluor 546 Goat anti-mouse IgG2b (Thermo Fisher Scientific/Invitrogen, Waltham, MA, United States).

### Quantitative Fluorescent Western Blot Protein Expression Analysis

**FIGURE 2:**
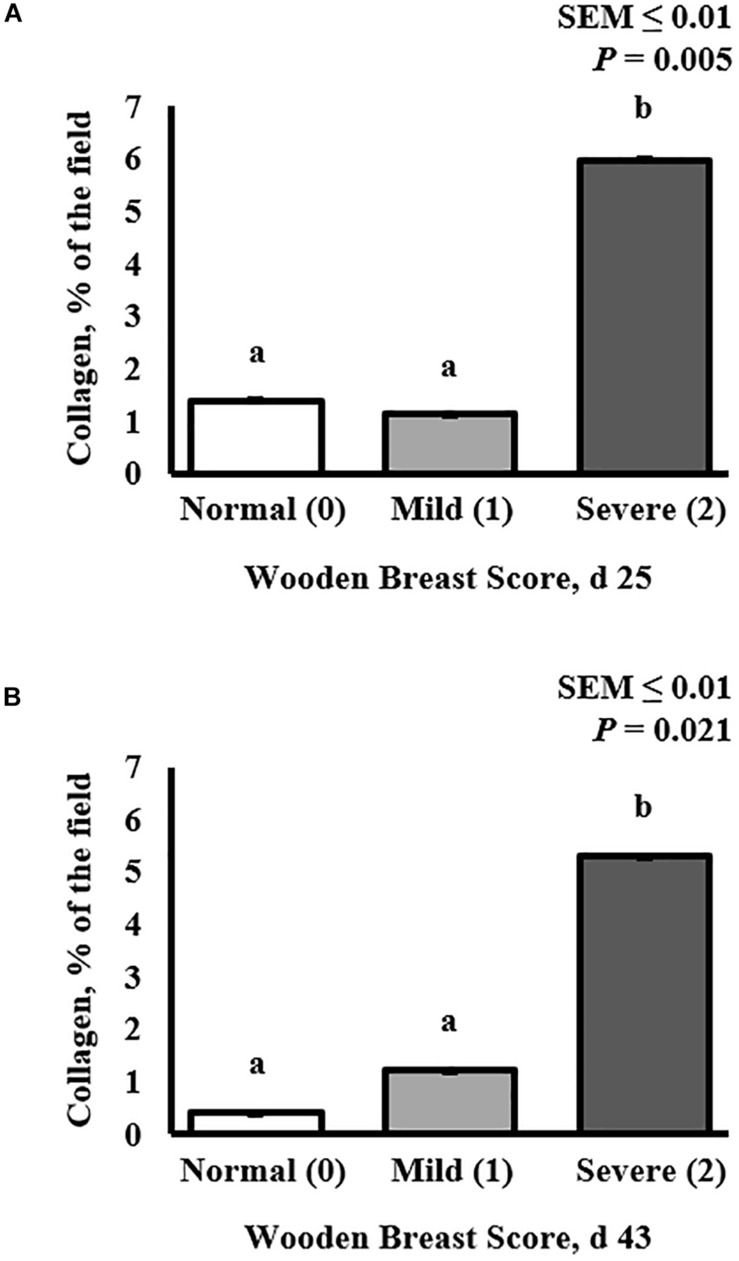
Effect of Wooden Breast (WB) on *pectoralis major* muscle collagen infiltration 43 days post-hatch in broiler chickens. **(A,B)** Demonstrate the increase in the proportion of collagen per digital image in muscles severely affected by WB on both day 25 (*P* = 0.005) and 43 (*P* = 0.021), respectively (*n* = 8 per score). ^a,b^Means with different superscripts differ *P* < 0.05.

Pectoralis major muscle tissue samples (∼250 mg) were placed in ice cold T-PER lysis buffer (Cat. No. 78510; Thermo Fisher Scientific) supplemented with a 2X final concentration of Halt protease and phosphatase inhibitor cocktail (Cat. No. 78441; Thermo Fisher Scientific). Samples were homogenized using a Qiagen TissueLyser II (Cat. No. 85300; Qiagen, Germantown, MD, United States) twice at 30 Hz for 2 min using the manufacturer’s instructions for homogenization. After homogenization, samples were centrifuged at 12,000 × *g* for 10 min. Supernatants were carefully removed and protein concentrations were determined using a Pierce BCA Protein Assay Kit (Cat. No. 23225; Thermo Fisher Scientific) with a NanoDrop One spectrophotometer (ND-ONEC-W; Thermo Fisher Scientific). Samples at 160 μg of total protein were mixed with lysis buffer to achieve a 20-μL final volume. Samples were then mixed with 1 μL of Cy5 dye from the Amersham QuickStain Protein Labeling Kit (Cat. No. RPN4000; GE Healthcare, Chicago, IL, United States) to stain total protein. Samples were incubated at room temperature in the dark for 30 min per the manufacturer’s instructions for the Amersham QuickStain Protein Labeling Kit. After, 4X Fluorescent Compatible Sample Buffer (Cat. No. LC2570; Invitrogen) and β-mercaptoethanol were added to each sample to achieve a final concentration of 1X sample buffer and 10 m*M* β-mercaptoethanol. Samples were vortexed, and then heated to 95°C and held for 3 min. Samples were loaded onto 4 to 20% gradient Criterion TGX precast midi gels (Cat. No. 5671094; Bio-Rad, Hercules, CA, United States) with Amersham ECL Plex Fluorescent Rainbow Markers (Cat. No. RPN851E; GE Healthcare) being added to the first and last lanes of each gel. Gels were electrophoresed at 80 V for 10 min and then 120 V for 60 to 65 min (until the dye front reached the bottom of the gel) in a Criterion Electrophoresis Midi Vertical Cell (Cat. No. 1656001; Bio-Rad). After electrophoresis, gels were transferred to low-fluorescent polyvinylidene fluoride (PVDF) membranes from a Trans-Blot Turbo RTA Midi LF PVDF Transfer Kit (Cat. No. 1704275; Bio-Rad) using a Trans-Blot Turbo Transfer System (Cat. No. 1704150; Bio-Rad) per the manufacturer’s instructions. Membranes were then blocked for 1 h at room temperature using Intercept (TBS) Blocking Buffer (Cat. No. P/N: 927-60001; LI-COR Biosciences, Lincoln, NE, United States). After blocking, membranes were incubated in anti-Type 1, α1 Collagen (Cat. No. sc-8784-R; SCB) primary antibody diluted 1:500 in Intercept T20 (TBS) Antibody Diluent (Cat. No. P/N:927-65001; LI-COR) overnight (∼16 h) at 4°C. The following morning, membranes were washed three times for 5 min each in *tris*-buffered saline + 0.01% Tween 20 (TBST). Membranes were incubated in AlexaFluor Plus 555 Goat anti-Rabbit IgG (H+L) Highly Cross-Absorbed Secondary Antibody (Cat. No. A21428; Thermo Fisher Scientific) diluted 1:5,000 in Intercept T20 (TBS) Antibody Diluent at room temperature for 1 h. Membranes were then washed three times for 5 min each in TBST and allowed to air dry for 3 h in a dark room. Dried membranes were imaged using an Amersham Imager 600 (Cat. No. 29083461; GE Healthcare) using the fluorescent settings for green/Cy3 (collagen, and green fluorescent protein ladder markers), and red/Cy5 (total protein and red fluorescent protein ladder markers) channels for 5 and 4 s, respectively. Fluorescent band intensity for collagen and total protein were quantified using Image Quant TL 8.1 software (Cat. No. 29000737; GE Healthcare). Collagen protein expression was first normalized to total protein on a per lane (individual bird) basis and then set relative to the mean WB score 0 expression (Figure 2).

### Statistical Analysis

Statistical analysis was performed using the GLIMMIX procedure of SAS (PC version 9.4, SAS Inst. Inc., Cary, NC, United States). For all data analysis, WB score served as the fixed effect and the Satterthwaite adjustment was used to correct degrees of freedom with individual bird serving as the experimental unit. Bird BW and PM weight were tested as possible covariates for all independent variables and were found to be insignificant resulting in their exclusion from the model. Proportional data were analyzed using the events/experiments syntax with a binomial distribution and both continuous and proportional data were analyzed using an R-side covariance structure. All treatment means were separated using the PDIFF option and considered different when *P* ≤ 0.05. Tendencies for differences among treatment means were declared when 0.0501 ≤ *P* ≤ 0.10.

## Results

### Muscle Satellite Cell Population Heterogeneity

Heterogeneity of MSC populations in broilers with varying WB severity were assessed and are reported in Table 1 (day 25) and 2 (day 43). Heterogeneity and densities of the Myf-5+, MyoD+, and Pax7+ MSC in the PM of broilers harvested at 25 days post-hatch were similar among WB score (*P* > 0.10; Table 1). However, at day 43 post-hatch, there were considerable alterations in the Myf-5+, MyoD+, and Pax7+ MSC populations in PM of broilers with varying WB scores (Table 2). In 43-days-old broilers, as WB score increased the density of the Pax7+ MSC (*P* = 0.067) and relative density of MyoD+:Pax7+ MSC as a proportion of the total MSC population tended to increase (*P* = 0.085). Score 1 or mildly WB-affected birds also had increased densities of Myf-5+ (*P* = 0.092), MyoD+ (*P* = 0.03), Myf5+:MyoD+ (*P* = 0.046) compared with normal and severely affected (score 2) birds. In addition, the density of MSC expressing all 3 MSC markers (Myf-5+:MyoD+:Pax7+) was greater in muscles of mildly affected birds compared with unaffected and severely affected with WB (*P* = 0.048). The density and relative densities of the Myf5+:Pax7+ and MyoD+:Pax7+ MSC populations were unaltered by WB (*P* > 0.10). The relative density of Pax7+ MSC as a proportion of total DAPI+ and total myogenic cells was greater in severely affected broilers compared with mildly affected birds (*P* ≤ 0.042; Figure 1A). Densities of the non-myogenic Myf-5-:MyoD-:Pax7- populations were similar among WB scores (*P* = 0.236). However, the mildly affected (score 1) PM had lower proportions of total cells considered non-myogenic than muscles from unaffected or severely affected birds 43 days post-hatch (*P* = 0.05; Table 2).

### Collagen Infiltration and Collagen Protein Expression

**FIGURE 3:**
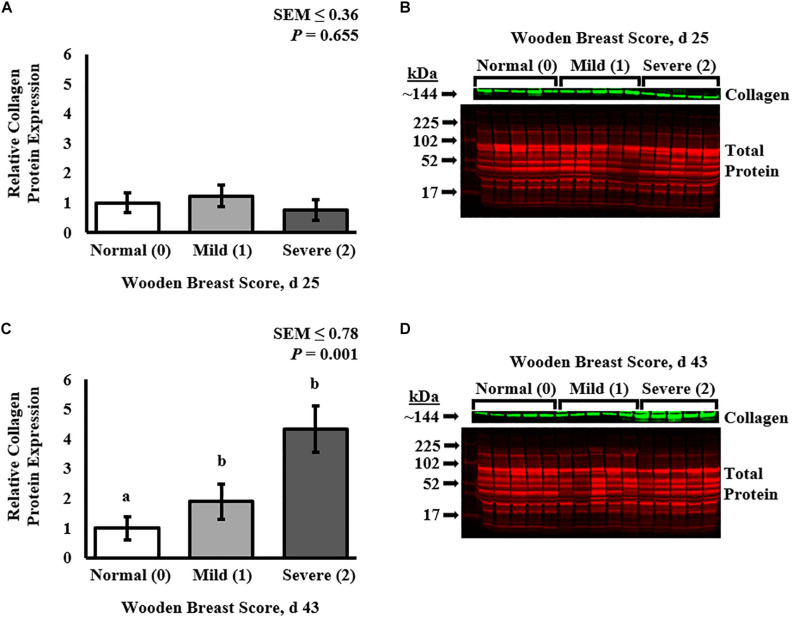
Effect of Wooden Breast (WB) on relative collagen protein expression in the *pectoralis major* muscle of broiler chickens at 25 and 43 days post-hatch using fluorescent Western Blot analysis. Collagen protein expression was first normalized to total protein on a per lane (individual bird) basis and then set relative to the mean normal (WB score 0) expression (*n* = 8 per score per d). At 25 days of age **(A,B)**, broiler PM muscle collagen protein expression was similar among all WB scores (*P* = 0.655). At day 43 **(C,D)**, collagen protein expression was increased in WB-affected PM muscles (WB scores 1 and 2) compared with normal (WB score 0) muscles (*P* = 0.001). ^a,b^Means with different superscripts differ *P* < 0.05.

**FIGURE 4:**
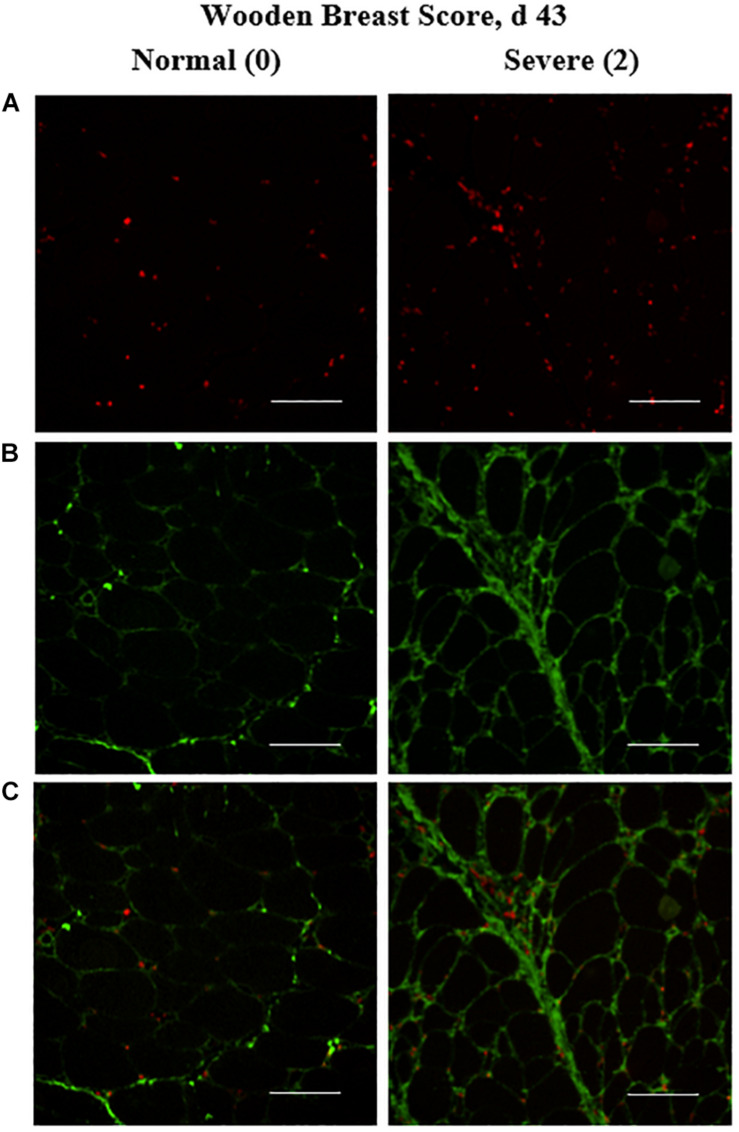
Effect of Wooden Breast (WB) on macrophage density (per mm^2^) and collagen infiltration into the *pectoralis major* (PM) muscle of broilers reared to 43 days of age (*n* = 8 per score per d). **(A)** Show representative cryohistological immunofluorescence staining images from normal (WB score 0) and severely WB-affected (WB score 2) PM muscles of broilers at 43 days of age for macrophages (red), **(B,C)** illustrate the location of the macrophages (red) in relation to the collagen (green) co-immunostaining located largely between myofibers. Scale bar = 100 μm.

Collagen infiltration into WB-affected PM muscle was assessed at both 25 and 43 days post-hatch in PM muscle cryosections by immunofluorescence staining and digital fluorescence microscopic analysis (Figures 1, 4) and quantitative results are displayed in Figure 2. At both ages, severely affected broilers (WB score 2) exhibited greater proportions of collagen per image compared with mildly affected and normal birds (*P* ≤ 0.021; Figures 1, 2). Relative collagen protein expression in PM tissue was also assessed at both days 24 and 43 in the same birds sampled for the cryohistology analysis using fluorescent Western Blotting (Figure 3). On day 25 post-hatch, relative collagen expression was similar among WB score (*P* = 0.655), while on day 43, birds affected with WB had increased collagen protein expression compared with unaffected broilers (*P* = 0.001).

### Muscle Macrophage Density

**FIGURE 5:**
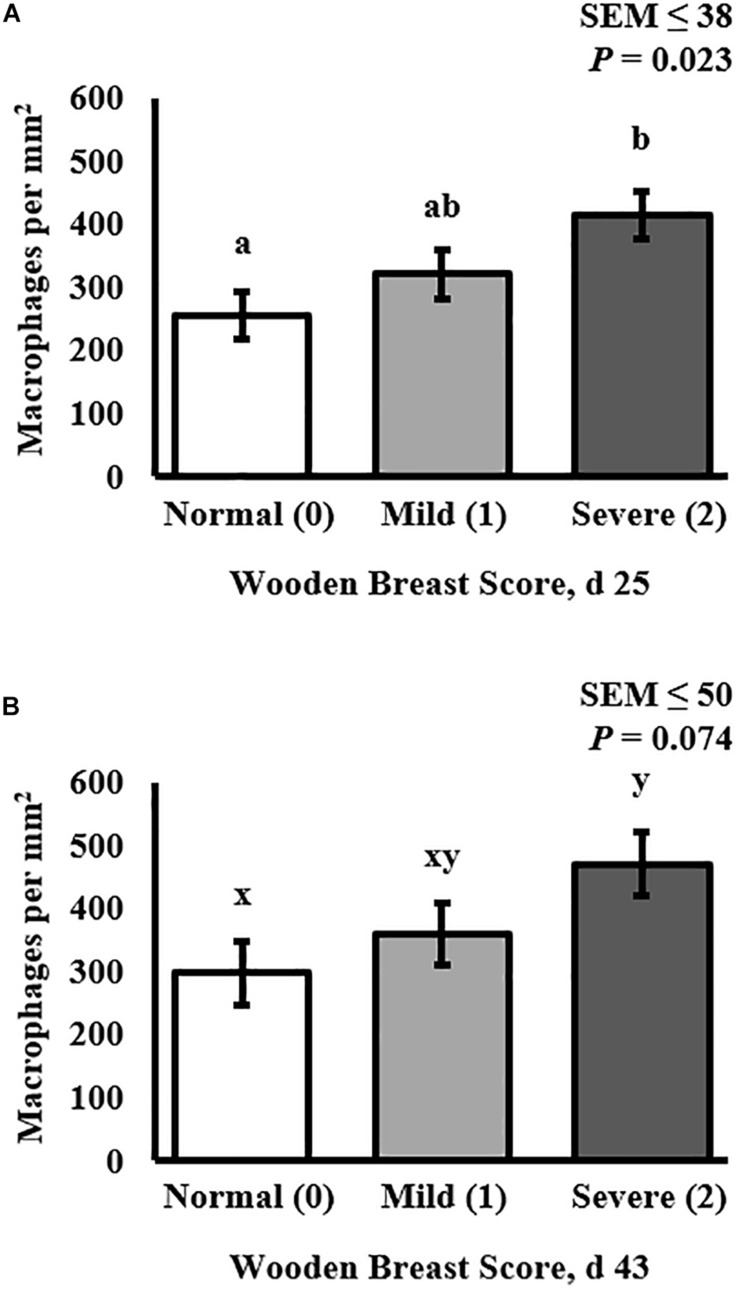
Effect of Wooden Breast (WB) on macrophage density (per mm^2^) in the *pectoralis major* (PM) muscle of broilers reared to 43 days of age (*n* = 8 per score per d). **(A)** Demonstrates that macrophage density increases as WB score increases in 25-days-old broilers (*P* = 0.023). **(B)** Demonstrates that macrophage density tended to increase as WB score increased in 43-days-old broilers (*P* = 0.074). ^a,b^Means with different superscripts differ *P* < 0.05.

The density of the total macrophage population (including both pro- and anti-inflammatory cells populations) in the PM of broilers was assessed by cryohistological and immunofluorescence analysis at both days 25 and 43 post-hatch in broilers with varying degrees of WB severity (Figure 4) and quantitative results are shown in Figure 5. The density of macrophages increased as WB score increased at both 25 (*P* = 0.023) and 43 (*P* = 0.074) days post-hatch (Figure 5).

## Discussion

The cellular and molecular mechanisms involved in the development of the broiler chicken WB myopathy are still not well-understood and the underlying cause has yet to be elucidated. Many different nutritional and management strategies have been aimed at eliminating the condition. Those strategies have included feed restrictions to slow growth rates, reductions in dietary nutrient density and specific nutrients, addition of antioxidants and chelated minerals, changes in electrolytes, changes in management such as restricting lighting and changing temperature conditions in the hatching and rearing facilities (Trocino et al., 2015; Wedekind et al., 2016; Chen et al., 2017; Kindlein et al., 2017; Livingston and Brake, 2017; Manangi et al., 2017; Meloche et al., 2018b,c,d). Yet, all these different nutritional and management strategies have failed to completely eliminate the WB myopathy in fast-growing, high-yielding commercial broilers.

The broiler industry’s inability to eliminate WB through post-hatch nutritional and management strategies combined with its widespread manifestation in a large proportion of the modern commercial broiler genetic lines grown globally suggests that selection of modern broilers over the last several decades for breast meat yield and feed efficiency placed inadvertent selection pressure on post-hatch hypertrophic growth of muscle fibers instead of pre-hatch muscle fiber hyperplastic growth. It is possible that this has contributed to post-hatch muscle tissue architecture with limited vascularity and oxygenation capacity creating a cellular environment that is simply incompatible with normal muscle growth and maintenance resulting in the WB myopathic phenotype. This theory is supported by recent work focused on exploring the differential transcriptomic and proteomic gene expression profiles in which dysregulation of various metabolic and muscle maintenance pathways have been observed (Mutryn et al., 2015; Abasht et al., 2016; Soglia et al., 2016, 2019; Kuttappan et al., 2017; Brothers et al., 2019; Greene et al., 2019).

The role of MSC function in the development of the WB phenotype is also not clear. However, the limited work conducted to date suggests that MSC function eventually becomes compromised in WB-affected broilers, leaving the rapidly growing PM improperly maintained, thus creating an environment in which collagen infiltration/fibrosis occurs (Velleman, 2015; Daughtry et al., 2017). The increases in mRNA for the MRF, MyoD and Myogenin, and the collagen cross-linking regulator, Decorin, in broilers with severe WB (Velleman and Clark, 2015) combined with the observation that MSC differentiation capacity is reduced as broilers age (Daughtry et al., 2017) support this view. Our previous findings that the mitotic activity of MSC populations and myofiber CSA distributions are significantly altered as WB scores increase further support the idea that MSC function is compromised in WB-affected broilers (Meloche et al., 2018a).

The WB myopathic phenotype in broilers has been largely characterized from a histopathological standpoint using paraffin histology, single antigen immunohistochemistry, and various traditional histological stains such as hematoxylin, eosin, and Masson’s trichrome with light microscopy methods. In the current study, our objective was to use a combination of cryohistological and immunofluorescence microscopy and quantitative protein expression techniques to expand the exploration of the cellular and molecular changes that occur in WB-affected broilers over time. Here, we characterized the heterogeneity of MSC populations expressing 3 MRF (Myf-5, MyoD, and Pax7) and quantified macrophage density, collagen infiltration, and collagen protein expression in normal, mildly affected, and severely affected broilers at 25 and 43 days of age. The use of experimental techniques such as cryohistology and multiplexed immunofluorescence staining as well as quantitative fluorescent Western Blotting to explore the WB myopathy at the cellular and molecular level is novel compared with current literature employing traditional paraffin histological analyses.

Here, no differences in the Myf-5, MyoD, and Pax7-expressing MSC populations were observed at day 25 in broilers differentially affected with WB (Table 1). This finding is in agreement with our previous study where the size of the total Myf-5+ and Pax7+ populations were similar in PM muscles from birds with WB scores of 0, 1, and 2 at day 25 post-hatch (Meloche et al., 2018a). On day 43, however, there were alterations in the Myf-5, MyoD, and Pax7-expressing MSC populations (Table 2). Interestingly, the Score 1 or mildly WB-affected birds had increased densities of Myf-5+, MyoD+, Myf5+:MyoD+, Myf-5+:MyoD+:Pax7+ MSC populations compared with WB scores of 0 and 2 (Table 2). The changes observed in this study at 43 days in the various Myf-5 and Pax7-expressing MSC populations among various WB scores are also similar to those observed in our previous work (Meloche et al., 2018a). The shifts observed in the MSC populations expressing MyoD at day 43 are in agreement with previous reports of increased MyoD mRNA transcripts in muscle severely affected with WB (Velleman and Clark, 2015). However, the reason for these shifts in the MSC growth kinetics that occur during the development of the WB myopathy are not clear. Perhaps in a mildly affected bird, the MSC are still in the process of trying to repair the damage and in birds of the same age that have already progressed to the severe phenotype this process has already ended. Based on these results, further investigation of the proliferation and differentiation capacity of MSC from WB score 0, 1, and 2 birds both *in vitro* and *in vivo* is warranted.

The quantitative increases in collagen infiltration in immunofluorescence-stained PM cryosections within both the endomysial and perimysial layers of connective tissue in WB-affected muscles observed in this study (Figures 1, 4) align with previous literature in which traditional paraffin histopathology methods were utilized to demonstrate this striking characteristic of the WB myopathy (Sihvo et al., 2014, 2017; Velleman and Clark, 2015). In addition, the increased relative collagen protein content of WB-affected muscle observed in 43-days-old broilers is supported by others’ work in which the *in vivo* collagen synthesis rates are upregulated in WB-affected broilers (Maharjan et al., 2019).

The increases in PM macrophage density as WB score increased are in alignment with previous qualitative work describing immune cell infiltration as a histopathological characteristic of the WB myopathy (Sihvo et al., 2014, 2017). We are unaware of other reports in which density of these resident phagocytic immune cells has been quantified in relation to WB severity over time. The major limitation of our macrophage analysis is the inability to distinguish between the pro- and anti-inflammatory macrophage populations due to the absence of commercially available antibodies reactive to these macrophage populations in chickens. Further characterization of these functionally divergent macrophage populations as well as their cell signaling secretory products is warranted.

Overall, the shifts in the MSC population MRF heterogeneity observed previously as well as in this study are novel and may indicate dysregulation of the MSC proliferation and differentiation processes in WB-affected muscles. Determining whether this apparent issue with MSC function is a symptom or cause of the WB myopathy, how local macrophages are involved, and what autocrine and paracrine cell signaling mechanisms may be driving this apparent inability to maintain rapidly growing muscles in today’s high-yielding, commercial broiler chickens will require further investigation.

## Data Availability Statement

All datasets generated for this study are included in the article/supplementary material..

## Ethics Statement

The animal study was reviewed and approved by the Auburn University Institutional Animal Care and Use Committee under Protocol No. 2016-2829.

## Author Contributions

LK, TF, SV, VN, JF, KM, and JS conceptualized the studies and contributed to the scientific discussion. TF and JS conducted the immunofluorescence analysis. TF wrote the original draft of the manuscript. JF, LS, and JS conducted the fluorescent Western Blot protein quantification. JS oversaw all experiments and revised the manuscript.

## Conflict of Interest

The authors declare that the research was conducted in the absence of any commercial or financial relationships that could be construed as a potential conflict of interest. Mention of trade names or commercial products in this publication is solely for the purpose of providing specific information and does not imply recommendation or endorsement by Federal University of Rio Grande do Sul or Auburn University.
